# Head circumference percentiles in Indian children with Down syndrome

**DOI:** 10.3389/fped.2025.1563501

**Published:** 2025-04-28

**Authors:** Harvinder Kaur, Anil Kumar Bhalla, Inusha Panigrahi, Rupinder Kaur, Neha Sudhera

**Affiliations:** ^1^ Child Growth & Anthropology Unit, Department of Pediatrics, Postgraduate Institute of Medical Education & Research (PGIMER), Chandigarh, India; ^2^ Genetics & Metabolic Unit, Department of Pediatrics, Postgraduate Institute of Medical Education & Research (PGIMER), Chandigarh, India

**Keywords:** percentiles, trisomy 21, microcephaly, small head, northwest India

## Abstract

This study aimed to construct age- and sex-specific growth percentiles for head circumference (HC) that can be used as a reference for Indian children with Down syndrome (DS). Over 24 years, following a mixed-longitudinal growth research design, 2,327 head circumference measurements were performed on 1,125 (boys: 752, girls: 373) children with DS karyotypically proven as cases of free trisomy 21 who were aged <1 month to 10 years, following a standardized anthropometric technique. A steady increase in the mean head circumference of male and female children with DS was noted. Boys with DS had significantly larger HCs than girls. Our study showed that 12.9% of Down syndrome cases had normal head circumference, 27.2% had small heads, and the majority, 59.9%, had microcephaly. Head circumference percentiles for boys and girls with Down syndrome were constructed for ages <1 month to 10 years. There is a need to monitor the growth of children with Down syndrome using population-specific and specialized growth charts. The age- and sex-specific head circumference growth percentiles presented for Indian children with Down syndrome can be used for growth monitoring and inter-population comparison.

## Introduction

A small head (microcephaly) and a broad head (brachycephaly) are universally accepted as diagnostic criteria of Down syndrome (DS). In addition, growth retardation and compromised development of the brain along with several other features (sloping forehead, flat occipital bone, etc.) are frequently observed in these patients. While there have been studies examining the growth of children with DS in terms of body weight and height (1–4), published longitudinal information on the growth of head circumference (HC) is lacking worldwide. In the Indian context, a recent study by Priya et al. (5) and some information based on pilot research (6, 7) has been published.

Because the growth patterns of children with DS differ biologically from those of the normal population, as do the growth patterns of patients with DS in different population groups/races, using growth curves/charts developed for a specific racial/ethnic group may not be appropriate for making precise diagnoses and quantifying the extent of auxological insults experienced by these patients. Given the critical role that early identification of growth deviations plays in managing health outcomes, it is essential to have population-specific growth charts that reflect the unique genetic, environmental, and socioeconomic factors influencing the growth of children in different regions. Early identification of a range of pathologies such as microcephaly, intellectual disability, growth retardation, and delayed neurodevelopment can be best made by using syndrome-specific charts developed for patients belonging to the same population.

This study contributes to clinical practice by providing the first longitudinal, age- and sex-specific growth percentiles for head circumference in Indian children with DS. These data can help pediatricians, genetic counselors, and healthcare providers identify abnormal growth patterns early, allowing timely intervention. Furthermore, it will serve as a reference for assessing how children with DS from northwestern India grow relative to international standards and other ethnic groups, highlighting potential regional disparities that may affect health interventions.

## Material and methods

The sample for this serial study conducted over a 24-year period (August 1994–November 2018) consisted of 1,125 (boys: 752, girls: 373) children with Down syndrome aged <1 month to 10 years, who were karyotypically proven as cases of free trisomy 21. Patients with translocations or mosaicism were excluded from the study sample. These children hailed from the northwestern parts of India and were enrolled from the “Genetic Clinic” of the Department of Pediatrics. The clinical and demographic characteristics of the study participants have been published elsewhere (1).

The study protocol was approved by the Institutional Ethics Committee of Postgraduate Institute of Medical Education & Research (PGIMER). Prior to their enrollment, informed consent to participate in this study was obtained from the parents of these children. As the study spanned several years, the authors ensured compliance with ethical standards, ensuring participant rights and maintaining the confidentiality of the data throughout the extended research period. Various safeguards were implemented: informed consent was reaffirmed periodically, all data collected during the study were kept strictly confidential, patient identities were anonymized, and the data were coded to prevent the identification of individual participants.

The head circumference of each child was measured at the Growth Clinic to the nearest 0.1 cm using a standardized anthropometric technique with a non-stretchable fiberglass tape at monthly age intervals (time tolerance ±3 days) during the first year of life, at 6-month age intervals (time tolerance ±15 days) from 1 to 5 years of age, and at yearly intervals (time tolerance ±1 month) thereafter, following a mixed-longitudinal growth research design. The head circumference measurement was taken by a trained anthropometrist, and another person helped to gently restrain uncooperative young children while taking their measurements.

The Kolmogorov–Smirnov test was employed to check the normality of the data gathered from all patients with DS. As our data were normally distributed, the mean and standard deviation (SD) for head circumference at each age among the children with DS were calculated. A total of nine age- and sex-specific percentiles (i.e., 3rd, 5th, 10th, 25th, 50th, 75th, 90th, 95th, and 97th) were obtained for head circumference at each age.

The percentiles were calculated using the formula

n=(P×N)/100

where *P* denotes the percentile, *n* indicates the number, and *N* represents the total population count.

An unpaired *t*-test was applied to quantify the magnitude of sex differences. Statistical analyses were carried out using SPSS version 20 (SPSS Inc., Chicago, IL, USA).

Children with DS with a head circumference of ≤3SDS (standard deviation score) were categorized as having microcephaly, those with a head circumference between ≤2SDS and ≥3SDS as having a small head, and those with a head circumference between −2SDS and +2SDS as having a normal head (8, 9). For the same specified purpose, normal reference data for head circumference from the WHO (10) were used for comparison up to 5 years of age. Beyond 5 years of age, the International and Interracial graphs published by Nellhaus (11) were used. The MedCalc (12) calculator was used to compare the mean values of our children with DS with the mean values of normal children. A *p*-value <0.05 was considered significant.

## Results

A total of 2,327 head circumference measurements were taken from 1,125 children with DS over 24 years. A steady increase in the mean head circumference of both male and female children with DS was found throughout the study period. Except for having a smaller head circumference at 4 months, the boys with Down syndrome had a larger head circumference than the girls, with statistically significant differences (*p* < 0.05) at most age levels (Table 1). The mean increase in head circumference of the boys with DS (9.8 cm) was greater than that of the girls (8.43 cm) during the first year of life. Although reduced in magnitude, the mean increase was also greater in the male patients with DS (1.92 cm) than in their female counterparts (1.38 cm) during the second year of life. During the third year of life, the increase in head circumference was 1 cm among both male and female subjects with DS. The mean percent increase in HC from <1 month to 10 years was 47.9% (15.5 cm) in the boys and 40.9% (13.6 cm) in the girls.

**Table 1:** Mean (SD) and sex differences for head circumference (cm) in male and female children with Down syndrome.

Age	Down syndrome (boys)		Down syndrome (girls)		Sex differences
	*N*	HC (cm), mean ± SD	*N*	HC (cm), mean ± SD	
1 month	10	34.4 ± 1.57	11	33.6 ± 2.10	0.380
2 months	28	36.3 ± 1.86	11	34.8 ± 1.28	0.018^a^
3 months	19	36.4 ± 2.28	13	34.9 ± 1.25	0.04^a^
4 months	22	37.2 ± 1.97	12	37.5 ± 2.46	0.70
5 months	22	38.9 ± 1.77	11	37.9 ± 1.40	0.09
6 months	31	39.4 ± 2.07	18	38.2 ± 2.34	0.08
7 months	32	39.9 ± 1.87	15	38.8 ± 1.58	0.05
8 months	28	40.8 ± 1.89	13	38.8 ± 1.46	0.006^b^
9 months	17	41.5 ± 1.13	13	39.8 ± 0.77	0.000^c^
10 months	25	41.6 ± 1.04	13	40.3 ± 1.44	0.003^b^
11 months	21	41.6 ± 1.74	12	41.6 ± 2.39	1.00
1 year	132	42.2 ± 1.62	74	41.7 ± 1.94	0.06
1.5 years	113	43.2 ± 1.57	51	42.2 ± 1.75	0.001^b^
2 years	123	44.1 ± 1.22	56	43.1 ± 1.27	0.000^c^
2.5 years	105	44.7 ± 1.70	56	43.3 ± 1.42	0.000^c^
3 years	111	44.9 ± 1.33	54	43.9 ± 1.55	0.000^c^
3.5 years	91	45.3 ± 1.46	35	44.3 ± 1.03	0.001^b^
4 years	107	45.4 ± 1.98	38	44.6 ± 1.43	0.024
4.5 years	74	45.7 ± 1.47	32	44.9 ± 1.56	0.019^a^
5 years	111	46.0 ± 1.26	36	45.1 ± 1.27	0.000^c^
6 years	92	46.3 ± 1.40	44	45.3 ± 1.27	0.000^c^
7 years	73	46.8 ± 1.21	29	46.0 ± 1.51	0.007^b^
8 years	65	46.8 ± 1.45	19	46.2 ± 1.34	0.084
9 years	52	47.3 ± 1.55	19	46.5 ± 1.13	0.030^a^
10 years	107	47.8 ± 1.48	31	46.8 ± 1.29	0.017^a^

HC, head circumference.

^a^ *p* < 0.05.

^b^ *p* < 0.01.

^c^ *p* < 0.001.

Compared to their normal WHO Multicentre Growth Reference Study (MGRS) counterparts (10), our children with DS had a significantly (*p* < 0.001) smaller head circumference up to 5 years of age (Supplementary Table). Because comparative reference data for the head circumference of Indian children with DS are not available beyond 5 years, inter-population comparisons could not be made.

Microcephaly was found in 59.9% (*n* = 1,394) of our study children, while 27.2% (*n* = 633) had a small head. Interestingly, a normal head circumference was detected in 12.9% (*n* = 300) of our children with DS. Head circumference-for-age percentiles are presented in Tables 2, 3.

**Table 2:** Head circumference-for-age percentiles for boys with Down syndrome.

Age	Birth to 10 years (percentiles)								
	3rd	5th	10th	25th	50th	75th	90th	95th	97th
1 month	32.0	32.0	32.0	32.9	34.2	36.0	36.5	—	—
2 months	32.0	32.5	33.3	35.2	36.5	37.3	39.2	39.5	—
3 months	34.0	34.0	34.4	34.6	35.7	37.0	38.8	—	—
4 months	32.9	32.9	33.0	36.5	37.4	38.7	39.3	39.9	—
5 months	35.8	35.8	36.2	37.6	39.0	40.3	41.2	42.5	—
6 months	36.4	36.4	36.5	38.2	39.4	40.4	41.0	43.9	—
7 months	36.0	36.5	37.1	38.1	40.0	41.0	42.5	43.3	—
8 months	37.0	37.5	38.2	39.2	40.5	42.3	43.2	44.3	—
9 months	39.0	39.0	40.0	40.9	41.5	42.0	43.2	—	—
10 months	39.9	39.9	40.1	40.9	41.6	42.3	43.0	43.8	—
11 months	39.4	39.4	39.4	40.0	41.3	43.1	44.0	44.9	—
1 year	38.8	39.2	40.0	41.0	42.2	43.2	44.0	45.0	45.7
1.5 years	40.0	40.6	41.6	42.5	43.2	44.2	45.5	45.6	46
2 years	41.9	42.0	42.5	43.2	44.1	45.0	45.6	46.0	46.6
2.5 years	41.6	41.8	42.5	43.1	44.8	46.0	46.7	47.5	47.5
3 years	42.8	42.9	43.0	44.0	45.0	46.0	46.9	47.1	47.3
3.5 years	42.6	43.0	43.5	44.3	45.0	46.2	47.3	47.7	48.6
4 years	42.6	43.0	43.9	44.5	45.3	46.5	47.3	48.0	48.4
4.5 years	42.7	42.8	44.0	44.9	45.6	46.8	47.5	48.0	48.2
5 years	43.7	44.3	44.4	45.2	46.0	46.8	47.7	48.0	48.3
6 years	43.5	43.8	44.3	45.2	46.4	47.3	48.0	48.6	48.9
7 years	43.9	44.3	45.1	46.1	46.8	47.7	48.5	48.6	48.9
8 years	44.2	44.5	44.9	45.6	47.0	48.0	49.0	49.4	49.5
9 years	44.4	45.0	45.5	46.1	47.0	48.4	49.3	50.3	51.2
10 years	44.5	44.6	46.1	46.9	47.8	48.7	49.5	50.3	51.2

**Table 3:** Head circumference-for-age percentiles for girls with Down syndrome.

Age	Birth to 10 years (percentiles)								
	3rd	5th	10th	25th	50th	75th	90th	95th	97th
1 month	31.2	31.2	31.2	32.0	33.7	35.0	37.6	—	—
2 months	33.0	33.0	33.1	33.5	34.8	36.0	36.8	—	—
3 months	33.0	33.0	33.1	33.9	35.0	35.4	37.1	—	—
4 months	33.8	33.8	34.1	35.1	37.2	39.4	41.6	—	—
5 months	35.6	35.6	35.6	37.5	38.0	38.5	39.9	—	—
6 months	33.0	33.0	34.2	37.0	38.5	40.0	40.6	—	—
7 months	35.5	35.5	36.6	37.5	38.5	39.9	41.3	—	—
8 months	36.3	36.3	36.3	37.7	39.4	40.1	—	—	—
9 months	38.0	38.0	38.4	39.1	40.0	40.5	40.6	—	—
10 months	38.6	38.6	38.6	39.1	39.9	41.4	42.8	—	—
11 months	39.4	39.4	39.4	40.4	41.2	41.5	—	—	—
1 year	38.4	39.0	39.6	40.4	41.6	42.9	44.0	45.8	46.8
1.5 years	38.4	38.6	39.2	41.3	42.3	43.5	44.6	45.0	45
2 years	40.1	41.1	41.7	42.2	42.8	43.9	44.8	45.0	45.5
2.5 years	40.5	40.6	41.2	42.3	43.3	44.2	45.0	45.3	46.8
3 years	40.6	40.7	42.0	43.3	44.0	44.7	45.9	46.2	47.8
3.5 years	42.1	42.8	43.1	43.6	44.4	45.0	46.0	46.5	46.5
4 years	41.3	42.7	42.8	43.6	44.5	45.8	46.5	46.8	47.8
4.5 years	41.4	41.4	43.2	44.2	45.0	46.0	46.8	48.0	—
5 years	42.7	42.9	43.5	44.3	44.9	46.3	47.0	47.1	47.4
6 years	43.0	43.5	43.7	44.4	45.2	45.9	47.0	47.8	48.7
7 years	42.5	43.1	44.0	45.0	46.0	47.2	48.0	48.3	—
8 years	44.5	44.5	44.6	45.3	46.0	47.5	48.2	—	—
9 years	44.6	44.6	45.0	45.7	46.4	47.0	48.2	—	—
10 years	44.6	44.6	44.9	45.8	46.8	47.4	48.9	—	—

## Discussion

**Figure 1:**
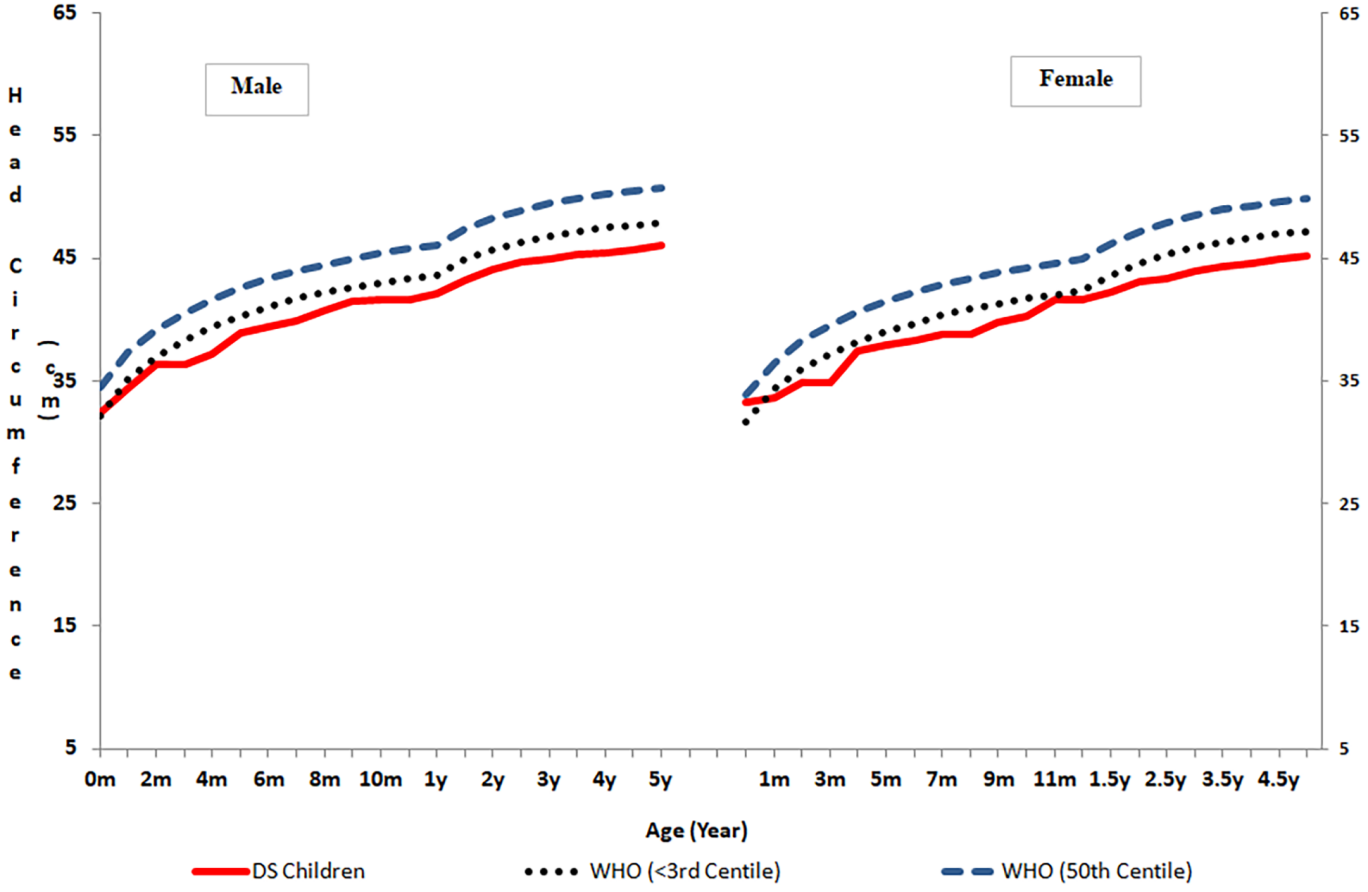
Comparison of head circumference in normal children and children with Down syndrome.

This is a single-center prospective study on the growth pattern of head circumference of karyotypically confirmed cases of DS from the northwestern regions of India. The age- and sex-specific percentile growth charts for head circumference presented in this article relate to serial observations made on children with DS from the age of <1 month to 10 years. A steady increase in the mean pattern of growth (50th percentile) of head circumference of the children with DS was recorded during the entire duration of this study. However, the scale of this increase was found to be greater up to the age of 3.5 years, after which it became substantially smaller up to the age of 10 years (Figure 1).

Patients with Down syndrome are characterized by impaired growth, and our results show that the mean head circumference of the children with DS was significantly smaller than that of healthy children. Furthermore, the mean increase in head circumference of the male (9.8 cm) and female (8.43 cm) children with DS during the first year of life substantially lagged behind the expected 12 cm gain in the first year of life (13). Despite experiencing much slower head growth than their healthy peers (10), our children with DS were able to complete 88% of their head growth by the age of 5. Interestingly, ∼13% of our children with Down syndrome had a normal head circumference, and ∼60% had microcephaly. Similar to our findings, normal head circumference in approximately 20% of Down syndrome cases has also been reported (14). However, Palmar et al. (15) observed that a staggering 50% of children with DS between the ages of 0 and 36 months had head circumferences similar to that of the general population range. Remarkably similar to our findings, Priya et al. (5) reported that approximately 25% of Indian children with DS had head circumferences between −2 and −3 SD.

As head circumference is considered a proxy for brain growth, head size, particularly in the early years of life, correlates with cognitive, motor, and adaptive development. The observed differences in head circumference growth in children with DS not only provide valuable insights into their physical development but also have important implications for neurodevelopmental outcomes. Children with Down syndrome with smaller head circumferences are known to exhibit developmental and motor delays (16). Hence, by tracking head circumference along with neurodevelopmental milestones, healthcare providers can better predict potential developmental delays and implement early interventions to support cognitive, motor, and adaptive skills.

Comparison of our data with other studies revealed that in Türkiye(17) and the United States (3), at <1 month to 10 years of age and in Brazil at 0–24 months of age (18), children with DS possessed larger HCs, as the 50th percentile plotted for our children with Down syndrome corresponded with their 10th percentiles. Similarly, the head circumferences of our girls with DS grew to approximately the 10th percentile when compared with the Down syndrome head circumference chart for the United Kingdom and the Republic of Ireland (19). Although small, the head circumferences of our children with DS were close to their Chinese peers (2), as the 50th percentile of our study children was close to their 25th percentile. At 3 years, the mean head circumference of our boys with DS was similar to their Egyptian counterparts (20). Surprisingly, when compared to their South Indian counterparts (6, 7), our children with DS from northwest India had smaller head circumferences. Because the study by Chandrasekhar and Ramachandran (6, 7) was conducted with a small population (*n* = 60), the discrepancies observed may have been an artifact of the small sample size, obscuring the true picture. The consistently lower placement on the growth curves plotted for the patients with DS in the present study than those of their Western and Chinese counterparts reveals that the growth of head circumference in our patients with DS remains impaired throughout as compared to their peers representing other racial groups. The significant inter-population variability observed in the head circumference growth of children with DS from northwestern India compared to children from other populations may be due to variability in their genetic make-up or availability of better healthcare, socioeconomic and environmental conditions, and sound nutritional and feeding practices. While all the participants in this study were diagnosed with free trisomy 21, there may be genetic diversity among children with DS in other studies. These differences highlight the importance of using population-specific growth charts and exploring the underlying causes of such variation to better understand the growth patterns of children with DS and how they may be influenced by these factors.

This study presents the first comprehensive set of age- and sex-specific longitudinal data on head circumference in children with DS of northwestern Indian origin. These growth percentiles are based on high-quality precision data acquired by a skilled anthropometrist and technician using a standardized anthropometric approach in karyotypically proven trisomy 21 cases with a known date of birth. The data provided can be used to monitor and assess the impact of different need-based interventions on the growth of Indian children with DS from northwest India. However, the findings may not reflect the head growth of children with DS from other population groups due to population-specific variability.

Notably, the study relies on international and interracial growth charts for children older than 5 years (11). Ideally, more current and population-specific reference data should have been utilized, as there is a significant gap in the literature regarding Indian-specific reference data for this age range. In addition, the study does not account for unmeasured confounders such as socioeconomic status, parental education, and access to healthcare. While the study excluded children with translocations or mosaicism, it did not consider other health conditions, such as concurrent cardiac or gastrointestinal disorders, that are often observed in children with DS.

Future research should aim to validate these findings in a larger, multi-center study including children from diverse regions of India, ensuring greater representativeness and broader applicability of the growth percentiles. This would help identify whether the observed trends hold true across different population groups and contribute to the development of national growth charts specific to Indian children with DS. In addition, future studies should consider integrating neurodevelopmental assessments to better understand the relationship between head circumference growth and cognitive or developmental outcomes of children with Down syndrome across diverse populations.

## Data Availability

The raw data supporting the conclusions of this article will be made available by the authors upon reasonable request. Requests should be directed to the corresponding author.
