# Supplementary material for: Head circumference percentiles in Indian children with Down syndrome
Source: Front Pediatr. 2025 Apr 28;13:1563501. doi: 10.3389/fped.2025.1563501 (PMC12066698; doi:10.3389/fped.2025.1563501)
Supplement: Supplementary file 1 [file Table1.docx]

**Supplementary Table: Comparison of Head Circumference in Normal and Down syndrome children**

| **Age** | **DS vs. normal (HC)** | | | |
| --- | --- | --- | --- | --- |
|  | **Male** | **CI** | **Female** | **CI** |
| 1m | 0.374 | (-1.37, .53) | 0.0011^b^ | (-2.53, 0.52) |
| 2m | 0.0001^c^ | (-4.156, 2.5) | 0.0001^c^ | (-4.33, -1.10) |
| 3m | 0.0001^c^ | (-3.87, 2.36) | 0.0001^c^ | (-3.87, -1.40) |
| 4m | 0.0001^c^ | (-3.96, 2.01) | 0.0010^b^ | (-3.56, -1.85) |
| 5m | 0.0001^c^ | (-3.57, 2.15) | 0.0001^c^ | (-4.69, -2.16) |
| 6m | 0.006^b^ | (-3.39,-- 1.67) | - | - |
| 7m | 0.0001^c^ | (-5.05,-3.51) | 0.0001^c^ | (-4.64, -2.88) |
| 8m | 0.0001^c^ | (-5.02, -3.58) | 0.0001^c^ | (-5.38, -3.57) |
| 9m | 0.0001^c^ | (-3.91, -2.73) | 0.0001^c^ | (-4.60, -3.60) |
| 10m | 0.0001^c^ | (-4.17, -3.18) | 0.0001^c^ | (-4.79, -3.24) |
| 11m | 0.0001^c^ | (-5.31, -3.55) | 0.0162^a^ | (-5.85, -1.04) |
| 1y | 0.0001^c^ | (-3.92, -3.46) | 0.0001^c^ | (-3.48, -2.73) |
| 1.5y | 0.0001^c^ | (-4.61, -3.69) | 0.0001^c^ | (-4.51, -3.15) |
| 2y | 0.0001^c^ | (-4.21, -3.81) | 0.0001^c^ | (-4.37, -3.79) |
| 2.5y | 0.0001^c^ | (-4.70, -3.51) | 0.0001^c^ | (-4.28, -3.14) |
| 3y | 0.0001^c^ | (-4.80, -4.38) | 0.0001^c^ | (-4.85, -4.14) |
| 3.5y | 0.0001^c^ | (-4.44, -3.62) | 0.0001^c^ | (-4.94, -4.05) |
| 4y | 0.0001^c^ | (-5.16, -4.54) | 0.0001^c^ | (-5.03, -4.20) |
| 4.5y | 0.0001^c^ | (-4.78, -3.95) | 0.0001^c^ | (-5.28, -3.93) |
| 5y | 0.0001^c^ | (-4.97, -4.48) | 0.0001^c^ | (-5.22, -4.36) |

Abbreviations: DS: Down syndrome; HC: Head Circumference

^a^p <0.05, ^b^p <0.01, ^c^p <0.001
